# Association of cord blood methylation with neonatal leptin: An epigenome wide association study

**DOI:** 10.1371/journal.pone.0226555

**Published:** 2019-12-18

**Authors:** Rachel Kadakia, Yinan Zheng, Zhou Zhang, Wei Zhang, Jami L. Josefson, Lifang Hou

**Affiliations:** 1 Division of Endocrinology, Ann and Robert H. Lurie Children’s Hospital of Chicago and Department of Pediatrics, Northwestern University Feinberg School of Medicine, Chicago, Illinois, United States of America; 2 Center for Population Epigenetics, Robert H. Lurie Comprehensive Cancer Center and Department of Preventive Medicine, Northwestern University Feinberg School of Medicine, Chicago, Illinois, United States of America; INIA, SPAIN

## Abstract

**Background:**

Neonatal adiposity is a risk factor for childhood obesity. Investigating contributors to neonatal adiposity is important for understanding early life obesity risk. Epigenetic changes of metabolic genes in cord blood may contribute to excessive neonatal adiposity and subsequent childhood obesity. This study aims to evaluate the association of cord blood DNA methylation patterns with anthropometric measures and cord blood leptin, a biomarker of neonatal adiposity.

**Methods:**

A cross-sectional study was performed on a multiethnic cohort of 114 full term neonates born to mothers without gestational diabetes at a university hospital. Cord blood was assayed for leptin and for epigenome-wide DNA methylation profiles via the Illumina 450K platform. Neonatal body composition was measured by air displacement plethysmography. Multivariable linear regression was used to analyze associations between individual CpG sites as well as differentially methylated regions in cord blood DNA with measures of newborn adiposity including anthropometrics (birth weight, fat mass and percent body fat) and cord blood leptin. False discovery rate was estimated to account for multiple comparisons.

**Results:**

247 CpG sites as well as 18 differentially methylated gene regions were associated with cord blood leptin but no epigenetic changes were associated with birth weight, fat mass or percent body fat. Genes of interest identified in this study are *DNAJA4*, *TFR2*, *SMAD3*, *PLAG1*, *FGF1*, and *HNF4A*.

**Conclusion:**

Epigenetic changes in cord blood DNA are associated with cord blood leptin levels, a measure of neonatal adiposity.

## Introduction

Adiposity at birth may be a predictor of obesity in childhood and adulthood.[1] Obesity has a current estimated prevalence of 17% among children aged 2–19 and has become increasingly challenging to treat once present.[2] Obesity related co-morbidities, such as type 2 diabetes and metabolic syndrome are now occurring earlier and more frequently in individuals with early life obesity.[3, 4] The etiology of obesity is complex, multifactorial, and includes genetic, nutritional, and environmental determinants. Researchers studying the developmental origins of health and disease have proposed that the intrauterine environment of the developing fetus contributes to adipose tissue deposition via fetal programming, suggesting that obesity risk is present before birth.[5, 6]

Epigenetics, the study of modifications to DNA that alter gene expression without changing gene sequence, is one mechanism contributing to the early life development of excess adiposity and future risk of an adverse metabolic phenotype.[7] Aberrant methylation of CpG dinucleotides in DNA is a type of epigenetic modification that can be both heritable and modifiable by one’s environment. Prenatal exposures, maternal pregnancy characteristics, and the intrauterine milieu can alter susceptibility of neonatal DNA to methylation, leading to changes in the child’s gene expression, gene regulation, and metabolic risk.[8, 9] Prior studies have demonstrated associations between maternal phenotypes and offspring DNA methylation, supporting the hypothesis that the intrauterine environment impacts fetal epigenetics.[10–12] Methylation patterns present at birth which are associated with newborn adiposity or later childhood obesity suggest that epigenetics may be partially responsible for the perinatal origins of obesity risk and predict future obesity. Identifying DNA methylation patterns associated with perinatal adiposity measures is one potential tool that can be used to detect high risk individuals early in life, a critical time point for early intervention before obesity develops.

Several research groups have identified epigenetic changes associated with newborn size; however many of these studies have used a targeted approach, only examining specific genes of interest such as *HIF3A* and *AHRR*.[13, 14] Studies that have previously taken an epigenome wide approach performed associations with indirect measures of newborn adiposity, such as birth weight.[15] Replication of specific epigenetic changes within independent cohorts are limited.

In this study, we evaluated the relationship between cord blood methylation patterns and markers of neonatal adiposity in a cohort of healthy, full-term infants born to mothers with normal glucose tolerance. Maternal hyperglycemia, even below the diagnostic threshold for gestational diabetes, is a well described risk factor for neonatal adiposity; therefore, elimination of this known confounder is key when investigating adiposity contributors.[16] We utilized an epigenome wide approach to examine differentially methylated regions in cord blood DNA and their association with newborn anthropometrics (birth weight, fat mass, and percent body fat) as well as cord blood leptin levels, a biomarker of neonatal adiposity with the aim to identify both novel and previously described epigenotype-phenotype relationships [8, 9, 11–15]. We hypothesize that cord blood epigenetic changes will be associated with measures of neonatal adiposity such as percent body fat, fat mass, and cord blood leptin, and provide insight into early life mechanisms of future obesity risk.

## Materials and methods

### Subjects

Our study population consisted of a cohort of 114 healthy maternal-neonatal pairs on whom cord blood DNA was available. Participants were recruited from 2011–2014 at a large academic medical center in Chicago, Illinois, USA as previously described in detail.[17] Women carrying a singleton pregnancy with normal glucose tolerance on a fasting two-hour 75g OGTT performed between 24 and 28 weeks gestation were eligible for the study.[18] Women were excluded if they had a history of greater than 3 term pregnancies, were on chronic medications such as glucocorticoids, insulin, or anti-hypertensives, or smoked during pregnancy, as these factors can be associated with excess or restricted growth.[19] Newborns were full-term and excluded if they required intensive care, were too ill to undergo body composition measurements within the first 24–72 hours of life, or had congenital anomalies, as some of these are independently associated with abnormal fetal growth. Cord blood was collected after birth by labor and delivery staff and processed within 30 minutes. Cord blood to be used for DNA extraction and future leptin assays was stored at -70°C until laboratory assays were performed. 115 maternal neonatal pairs were initially included in the study; one pair was excluded due to poor DNA quality. Of the 114 neonates included in the final analysis, 105 had body composition data available. This study was approved by the Northwestern University Institutional Review Board and each mother provided written informed consent for herself and her neonate at the time of study enrollment.

### Body composition measurements

Body composition measurements of each neonate occurred between 24–72 hours of life and were obtained in duplicate by one of two trained examiners. Length was obtained using a hard-surface measuring board. Measurements were recorded to the nearest 0.1cm, performed in duplicate, and the results averaged for the final research measure. Weight and adiposity measurements were obtained by method of air displacement plethysmography (PeaPod, Cosmed, Rome, Italy), a noninvasive, nonuser dependent modality that has been validated in comparison with deuterium dilution in full-term infants between ages 0.4–21.7 weeks and of weight 2–8 kg.[20, 21] To measure weight, the infant was undressed and placed on the calibrated PeaPod scale and weight was recorded to the nearest 0.0001 kg. Next, the infant was placed inside the PeaPod volume chamber for two minutes to determine body volume. Density was calculated after which age- and sex-specific fat-free mass density values were used to determine absolute fat-free mass and fat mass. Percent body fat was subsequently calculated from these values.[21]

### Laboratory measurements

Samples for leptin were batched and measured in duplicate with a radioimmunoassay kit (Millipore Corp, Billerica, MA, USA). The inter- and intra-assay coefficients of variation for leptin were 3.7–5.9% and 3.0–4.0%, respectively.

DNA was purified from neonatal cord blood using an Autopure LS Automated DNA Purification System with Autopure reagents (Autogen, Inc., Holliston, MA). The purified DNA samples were stored under -20°C. DNA quality and quantity were assessed by Nanodrop (ThermoFisher Scientific, MA, USA). Bisulfite conversion was performed on 500 ng of DNA using the EZ DNA Methylation Kit (Zymo Research, CA, USA). Methylation levels were measured using the Infinium HumanMethylation 450K Beadchip array (Illumina, Inc. CA, USA), which targets ~486,000 CpG sites, in 114 samples that passed DNA quality testing. Samples were randomly plated on each chip with regard to neonatal sex. BeadChips were scanned with an Illumina iScan and analyzed using Illumina GenomeStudio software. All experiments were conducted following manufacturer protocols in the Genomics Core Facility at the Center for Genetic Medicine at Northwestern University.

### DNA methylation data processing

Raw Illumina IDAT data were preprocessed per previously published methodology.[10] Briefly, one DNA sample containing more than 5% of CpG probes with detection p-values greater than 0·01, as well as 191 CpG probes that were not detectable (detection p-value > 0·01) in more than 5% of samples were removed during quality control. We further removed 65 built in SNP probes, 3,091 non-CpG probes, 36,535 probes containing proximal SNPs, and 11,648 probes on sex chromosomes. Signal intensities of the filtered probes were corrected for background noise and channel color bias. There were 434,506 CpG sites used in the final analysis. Methylated and unmethylated intensities were then quantile-normalized and corresponding β values were calculated (i.e., the proportion of methylated probe intensity out of total intensity). The function *ComBat* in R package *sva* was implemented to the normalized β values to adjust for potential batch effects and then the function *sva* in the same R package was applied to generate surrogate variables that were used to account for other unwanted variations in the data including the confounding effects of cell type heterogeneity on methylation profiles.[22]

### Bioinformatics and statistical analysis

We conducted an epigenome wide association study using previously described methodology.[23] Briefly, linear regression models were used with neonatal adiposity measures as dependent variables and methylation as the variable of interest. Models were adjusted for maternal age at delivery, race, gestational age in days, and infant sex, as these variables can affect neonatal body composition. Two surrogate variables generated by *sva* were also adjusted. An adjusted p value (FDR) < 0.05 following the Benjamini–Hochberg procedure was considered significant.[24]

We also examined the association of percent body fat, fat mass, and log_10_-transformed cord blood leptin levels with differentially methylated regions (DMRs) using the R package *DMRcate*.[25] T-statistics of CpG sites from EWAS were smoothed by chromosome using a Gaussian kernel smoothing function with bandwidth λ = 1000 base pairs and scaling factor C = 2. DMRs were assigned by grouping significant CpG sites (FDR < 0·05). We used Stouffer’s method to compute combined FDR as the statistical inference for that region and the mean coefficients from the regression models summarizes the regional effect.[26] Within each DMR identified, given the identical sample size and similar distribution of methylation values across all the adjacent CpGs, we applied an inverse-variance weighting approach to compute the weighted mean of coefficients across the CpGs, weighted by the inverse of the corresponding standard error. The weighted mean coefficients were then re-expressed as percent changes in cord blood leptin levels with every 0.01 methylation β value increase. All analyses were conducted using R software (version 3.3.1).

### Regulatory elements

Transcriptional regulations can be controlled by complicated interactions between regulatory elements, such as histone modifications and DMRs. In order to further explore potential functional implications of differentially methylated regions, we used the Encyclopedia of DNA Elements (ENCODE) Project [27] to find regulatory elements that overlapped or were nearby the significant DMRs. We used DNase I hypersensitivity sites (DNase), transcription factor binding sites (TFBS), and annotations of histone modification ChIP peaks pooled across cell lines (data available in the ENCODE Analysis Hub at the European Bioinformatics Institute http://ftp.ebi.ac.uk/pub/databases/ensembl/encode/integration_data_jan2011/). The hg19 human assembly from Genome Browser was used to provide DMR location information.

## Results

Descriptive statistics of the study participants are shown in Table 1. The majority of infants had a birth weight that was appropriate for gestational age and percent body fat of the cohort was normally distributed. 61% of mothers were normal weight while 34% were overweight or obese. No mothers in this cohort smoked during pregnancy.

**Table 1:** Maternal and neonatal characteristics.

**Demographics (n = 114)**	
Maternal Race	61% White 39% Non-White
**Maternal Characteristics (n = 114)**	**Mean ± SD**
Age at Delivery	32 ± 3.8 years
Pre-Pregnancy BMI	25·0 ± 6.1 kg/m^2^
Underweight (BMI <18.5 kg/m^2^)	3·5%
Normal Weight (BMI 18.5–24.9 kg/m^2^)	61·4%
Overweight (BMI 25–29.9 kg/m^2^)	13·2%
Obese (BMI ≥30 kg/m^2^)	21·9%
OGTT Fasting Glucose	4·2 ± 0·3 mmol/L
OGTT 1-hour Glucose	6·5 ± 1·4 mmol/L
OGTT 2-hour Glucose	5·6 ± 1·1 mmol/L
**Neonatal Characteristics (n = 114, unless noted)**	**Mean ± SD**
Sex	53% Male
	47% Female
Gestational Age	39·5 ± 1 week
Birth Weight	3502 ± 515 g
Length	51·0 ± 2·3 cm
Fat Free Mass (n = 105)	2947 ± 374 g
Fat Mass (n = 105)	377 ± 169 g
% Body Fat (n = 105)	11 ± 3·8%
Cord Blood Leptin (n = 111)	10·7 ± 8·8 mcg/L

**Fig 1:**
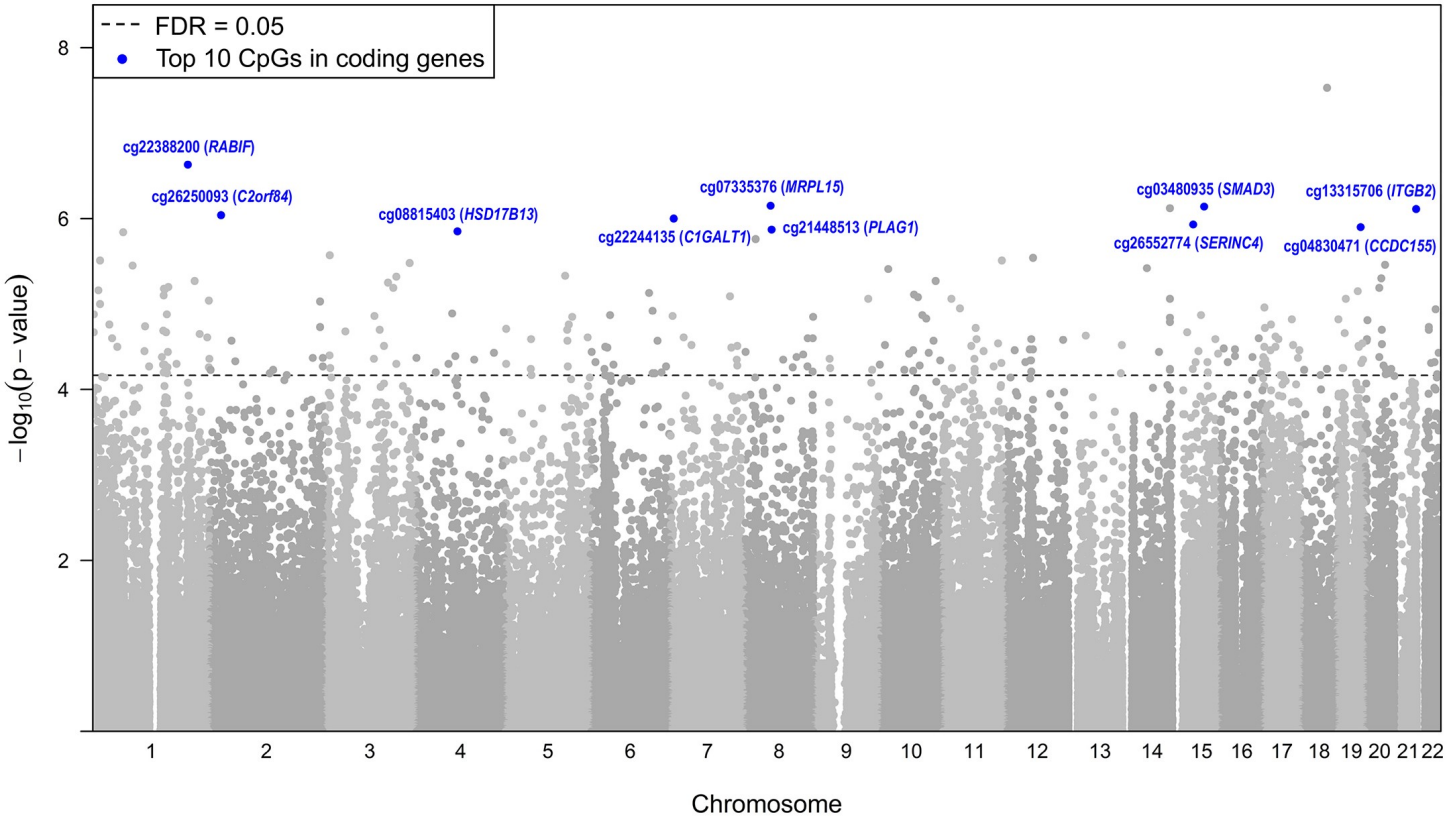
Manhattan plot. This manhattan plot depicts the CpG sites by chromosome that are associated with cord blood leptin. The dotted line defines a FDR adjusted significance level of < 0.05. The top 10 CpGs sites are identified within the figure.

We did not identify any associations between individual CpG sites or CpG gene regions and % fat, fat mass, or birth weight. When studying methylation of individual CpG sites, we found associations of 247 unique CpG sites with cord blood leptin, a marker of neonatal adiposity. Among them, 177 (72%) were negatively associated with leptin and 70 (28%) were positively associated with leptin (Fig 1, S1 Table). Using the DAVID Functional Annotation Tool (version 6.8) [28, 29], the genes targeted by the 177 negatively associated CpGs were enriched in lipid metabolic process (GO:0006629) (p = 0.0075), the genes targeted by the 70 positively associated CpGs were enriched in regulation of CD8-positive, alpha-beta T cell proliferation (GO:2000564) (p = 0.0077). The top 10 CpG sites are displayed in Table 2 with the remainder listed in S1 Table. All analyses were adjusted for maternal age at delivery, maternal race, neonatal sex, and gestational age.

**Table 2:** Differentially methylated CpG sites associated with cord blood leptin levels. This table includes the 10 most significant coding genes identified in our EWAS study. See S1 Table for a full list of significant CpG sites associated with cord blood leptin levels.

Gene	Gene Name	Chr	CpG site	Genomic region	% change in leptin^^^	P	FDR p*	Function
*RABIF*	RAB Interacting Factor	1	cg22388200	TSS1500	45.2	2.34E-07	0.02	Regulation of intracellular vesicular transport
*MRPL15*	Mitochondrial Ribosomal Protein L15	8	cg07335376	TSS1500	13.1	7.10E-07	0.02	Protein synthesis in mitochondria
*SMAD3*	SMAD Family Member 3	15	cg03480935	TSS200	-8.1	7.31E-07	0.02	Transcriptional modulator activated by TGF-beta; regulation of carcinogenesis
*ITGB2*	Integrin Subunit Beta 2	21	cg13315706	5’UTR	7.5	7.78E-07	0.02	Cell-surface protein that participates in cell adhesion and cell-surface mediated signaling; plays an important role in immune response
*C2orf84*	Family With Sequence Similarity 228 Member A	2	cg26250093	TSS1500	24.5	9.08E-07	0.02	Unknown
*C1GALT1*	Core 1 Synthase, Glycoprotein-N-Acetylgalactosamine 3-Beta-Galactosyltransferase 1	7	cg22244135	5’UTR	27.5	9.95E-07	0.02	Precursor for extended mucin-type O-glycans on cell surface and secreted glycoproteins
*SERINC4*	Serine Incorporator 4	15	cg26552774	TSS200	29.7	1.18E-06	0.02	Amino acid synthesis and interconversion
*CCDC155*	Coiled-Coil Domain Containing 155	19	cg04830471	TSS1500	-16.5	1.26E-06	0.02	Connection between nuclear lamina and cytoskeleton; essential for male and female gametogenesis
*PLAG1*	Pleiomorphic Adenoma Gene 1	8	cg21448513	5’UTR	-9.4	1.36E-06	0.02	Cell proliferation
*HSD17B13*	Hydroxysteroid 17-Beta Dehydrogenase 13	4	cg08815403	TSS200	-12.8	1.41E-06	0.02	Lipoprotein metabolism

^Percent change in leptin for every 0.01 increase in methylation beta value

*FDR adjusted p-value

TSS1500: 1500 bp from transcription start site; TSS200: 200 bp from transcription start site; 5’UTR: 5’ untranslated region

We also identified DMRs in 18 genes that were associated with cord blood leptin levels. The name and function of each gene, along with the location of each DMR is detailed in Table 3. Increased DMR methylation in 9 of these genes was negatively associated with cord blood leptin levels while hypermethylation in 9 genes demonstrated positive association with leptin. The function or proposed function of all genes is listed; however, 2 genes have not been well studied with regards to their role in disease development.

**Table 3:** Differentially methylated CpG regions associated with neonatal cord blood leptin.

Gene	Gene Name	Chr	DMR location	Genomic Region	# of CpG Sites in DMR	% change in leptin^	FDR p*	Function
*TRIM15 / TRIM10*	TRIM15: Tripartite Motif Containing 15 TRIM10: Tripartite Motif Containing 10	6	30127322–30130109	1stExon	19	-3.9	0.0114	Terminal differentiation of erythroid cells
*PSMB9*	Proteasome Subunit Beta 9	6	32825040–32826281	Body	15	-4.6	0.0114	Immunoproteasome involved in antigen processing to generate Class I binding peptides
*CEBPE*	CCAAT/Enhancer Binding Protein Epsilon	14	23586582–23589419	TSS1500	11	5.0	0.0114	Terminal differentiation and functional maturation of committed granulocyte progenitor cells
*DNAJA4*	DnaJ Heat Shock Protein Family (Hsp40) Member A4	15	78555907–78557584	TSS1500	14	-3.2	0.0114	Heat shock protein
*KIF13A*	Kinesin Family Member 13A	6	17985408–17986167	Body	3	-9.4	0.0221	Endosome positioning
*DMTN*	Dematin Actin Binding Protein	8	21915184–21917015	5'UTR	9	-4.9	0.0291	Actin binding and bundling protein that plays a structural role in erythrocytes
*RNF111*	Ring Finger Protein	15	59156878–59157857	Intergenic	6	4.5	0.0291	Induction of mesoderm during embryonic development
*MIR451B*	MicroRNA 451B	17	27187966–27189879	TSS1500	9	-4.7	0.0291	Noncoding RNA involved in mRNA stability and translation
*PIK3R5*	Phosphoinositide 3 Kinase Regulatory Subunit 5	17	8814822–8816266	5'UTR	6	-10.7	0.0313	Cell growth, proliferation, differentiation, motility, survival, and intracellular trafficking
*TMEM8A*	Transmembrane Protein 8A	16	432973–434356	Body	7	6.5	0.0313	Cell surface adhesion
*CCDC88B*	Coiled Cell Domain Containing 88B	11	64107158–64109823	Body	14	4.8	0.0313	Linking organelles to microtubules
*CMYA5*	Cardiomyopathy Associated 5	5	78985425–78986160	TSS200	12	2.1	0.0352	Anchoring protein Repressor of calcineurin mediated transcriptional activity
*MIR381HG*^¥^	Large intergenic non-coding RNA	14	101512592–101514051	TSS1500	9	-2.3	0.0358	Noncoding RNA involved in mRNA stability and translation
*KANK2*	KN Motif and Ankyrin Repeat Domains 2	19	11305231–11306319	5'UTR	4	12.4	0.0358	Cytoskeleton formation and regulation of actin polymerization
*TFR2*	Transferrin Receptor 2	7	100230781–100231672	Body	4	6.2	0.0358	Cellular uptake of transferrin-bound iron
*TSPO2*	Translocator Protein 2	6	41010111–41010728	Intergenic	7	-2.2	0.0358	Cholesterol and porphyrin transport; RBC membrane transport
*SNORA54*^¥^ */ NAP1L4*	SNORA5: Small Nucleolar RNA H/ACA box 54 NAP1L4: Nucleosome Assembly Protein 1 Like 4	11	2985112–2986541	Body	5	0.8	0.0358	SNORA54: Noncoding RNA NAP1L4: Histone chaperone
*SPG7*	Paraplegin Matrix AAA Peptidase Subunit	16	89598305–89598950	Body	3	6.4	0.0358	Mitochondrial metalloproteinase that plays a role in membrane trafficking, intracellular motility, organelle biogenesis, protein folding, proteolysis

^Percent change in leptin for every 0.01 increase in methylation beta value and calculated as the inverse-variance weighted average of all CpGs.

*FDR adjusted p-value

^¥^Functions of these genes, especially as they relate to disease, are not well understood.

Hg19 Human Assembly was used to determine DMR locations.

TSS1500: 1500 bp from transcription start site; TSS200: 200 bp from transcription start site; 5’UTR: 5’ untranslated region; Body: Gene body; 1stExon: First exon.

**Fig 2:**
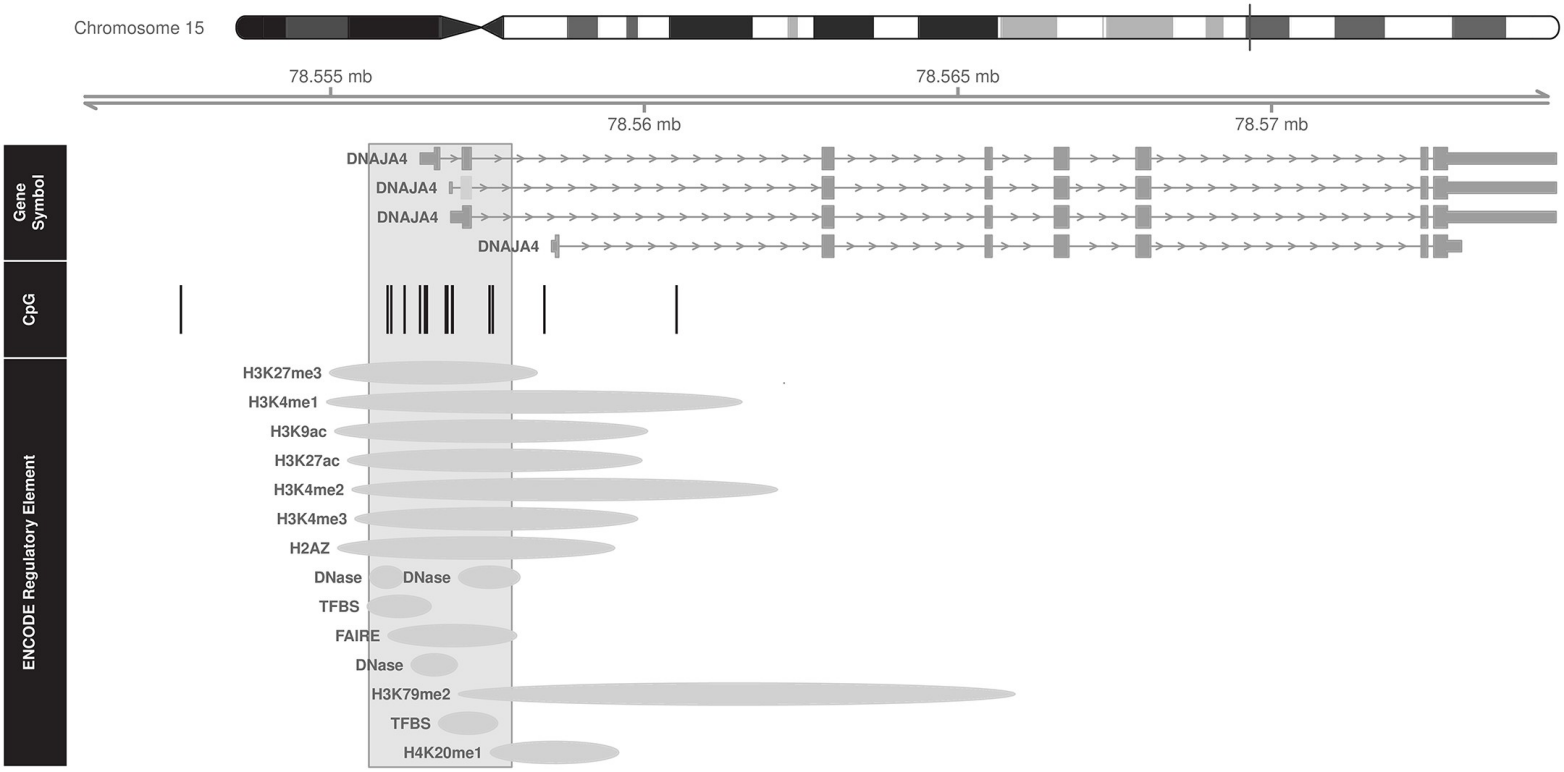
*DNAJA4*: Differentially methylated CpG sites associated with cord blood leptin levels and their relationship to overlapping regulatory elements. The black vertical bars within the gray shaded rectangle represent the differentially methylated CpG sites within the *DNAJA4* promoter that are associated with cord blood leptin levels. For every 0.01 increase in methylation in this region, neonatal leptin decreased by 3.2% (FDR adjusted p = 0.0114). The co-localization of *cis*-regulatory elements with the *DNAJA4* differentially methylated region are depicted as gray ellipses.

Increased methylation across 14 CpG sites in the DnaJ heat shock protein family (Hsp40) member A4 (*DNAJA4*) gene promoter (chromosome 15, position 78555907 to 78557584, hg19 genome assembly) was negatively associated with cord blood leptin levels (Fig 2). For every 0.01 increase in methylation in this region, neonatal cord blood leptin decreased by 3.2% (FDR adjusted p = 0.0114). Furthermore, multiple gene regulatory elements were found to overlap with the hypermethylated region (Fig 2). All CpG sites evaluated in the *DNAJA4* gene are displayed in S2 Table.

**Fig 3:**
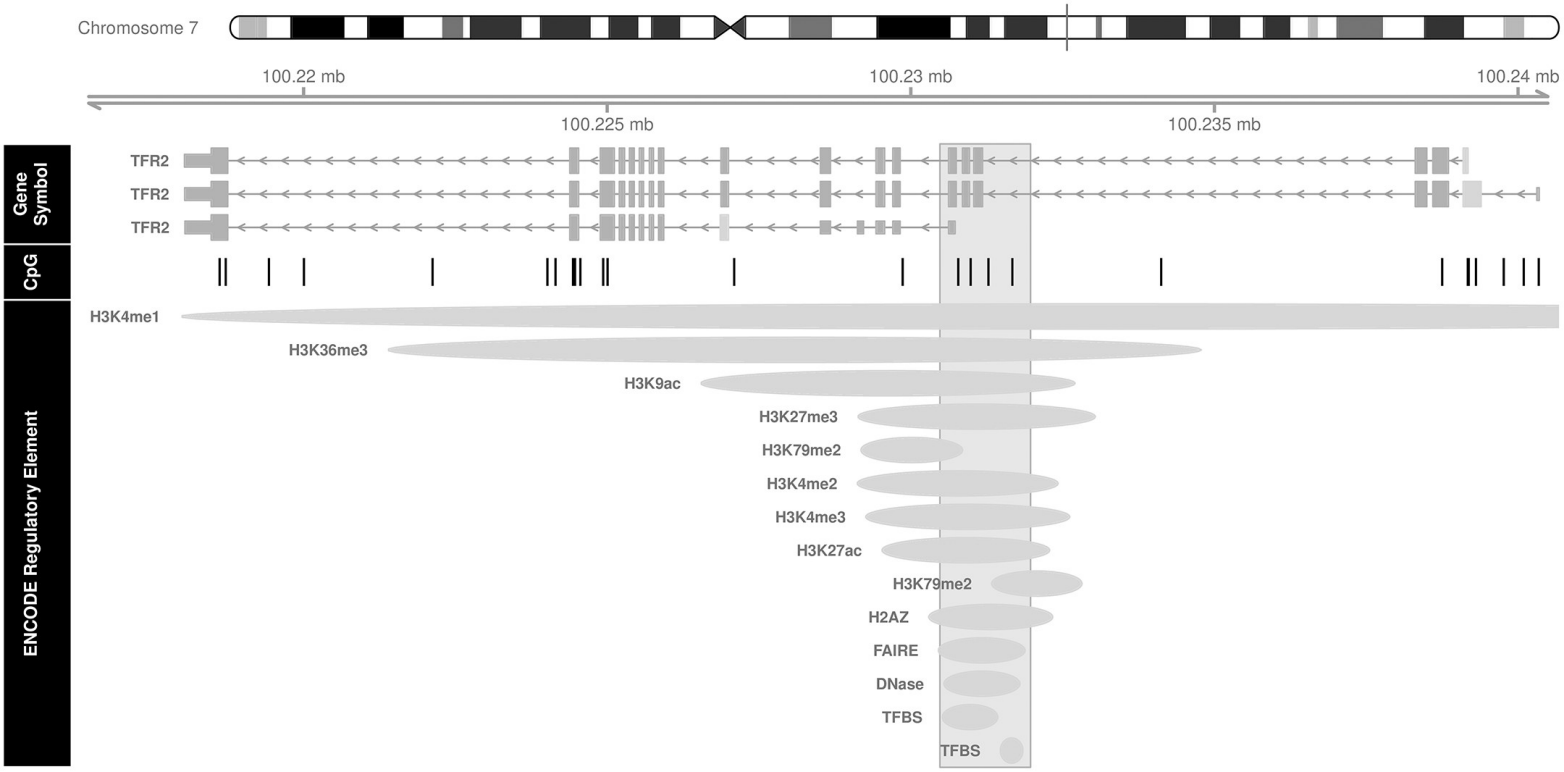
*TFR2*: Differentially methylated CpG sites associated with cord blood leptin levels and their relationship to overlapping regulatory elements. The black vertical bars within the gray shaded rectangle represent the differentially methylated CpG sites within *TFR2* that, together, are associated with cord blood leptin levels. For every 0.01 increase in methylation in this region, neonatal leptin increased by 6.2% (FDR adjusted p = 0.0358). The co-localization of *cis*-regulatory elements with *TFR2* differentially methylated region are depicted as gray ellipses.

A 0.01 increase in methylation across 4 CpG sites in the Transferrin receptor 2 (*TFR2*) gene (chromosome 7, position 100230781 to 100231672) was associated with a 6.2% increase in neonatal cord blood leptin (FDR adjusted p = 0.0358). These CpG sites are located at the promoter region of one *TFR2* transcript (Fig 3). All CpG sites evaluated in the *TFR2* gene are displayed in S2 Table. In addition, there are several regulatory elements that overlap with this differentially methylated region (Fig 3). We did not find any differentially methylated regions associated with anthropometric measures of neonatal adiposity.

## Discussion

This epigenome wide association study demonstrates a relationship between neonatal cord blood leptin levels and 1) 247 individual CpG sites and 2) differentially methylated regions of 18 genes. *SMAD3*, *PLAG1*, *FGF1*, *HNF4A*. *DNAJA4*, and *TFR2* are six genes identified in this study that have previously been reported in relation to tissue growth and adiposity. We chose to highlight these genes for their potential impact on adipose tissue development.

When evaluating associations between individual CpG sites and cord blood leptin levels, four genes are worth noting. *SMAD3* encodes for smad3, a signaling effector that mediates transforming growth factor-beta’s (TGF-beta) inhibition of adipocyte differentiation.[30] We identified that increased methylation at cg03480935 within *SMAD3* was associated with decreased cord leptin levels. This finding is unexpected as increased methylation is typically associated with decreased gene expression. In this case, potential decreased expression of *SMAD3* would lead to less inhibition of adipocyte differentiation and presumably higher leptin levels.

*PLAG1* is a transcription factor whose activation results in upregulation of target genes important for cell proliferation. *PLAG1* mutations are associated with lipoblastoma, a benign adipocytic tumor, suggesting its role in adipocyte growth and/or proliferation.[31] We found that increased methylation at cg21448513 within *PLAG1* was negatively associated with cord leptin levels (FDR adjusted p = 0.02). The increased methylation at cg21448513 in *PLAG1* may lead to decreased adipocyte proliferation and thus lower cord blood leptin levels.

*FGF1* is another gene clinically important for cell growth and described to have an adipogenic effect.[32] In a mouse model, high fat diet fed mice and the *ob*/*ob* mice had higher *FGF1* mRNA in their adipose tissue than mice fed a normal diet or lean control mice. [33] We identified that decreased methylation at a CpG site in the *FGF1* gene body (cg13724550, FDR adjusted p = 0.0412) is associated with leptin levels.

Lastly, *HNF4A* is another gene of interest. Increased methylation at a cg16121136 (FDR adjusted p = 0.0307) within the transcription start site was associated with lower leptin levels. Clinically, mutations in *HNF4A* can cause Mature Onset Diabetes of the Young, Type 1 (MODY1). Further study is necessary to understand the importance of altered methylation at these individual CpG sites on gene expression.

In our analysis of differentially methylated gene regions, DnaJ heat shock protein family (Hsp40) member A4 (*DNAJA4)* and Transferrin receptor 2 (*TFR2)* emerged as two interesting genes that have been previously reported in the literature to be associated with tissue growth and adipocytes, respectively.[34, 35] *DNAJA4* encodes for a heat shock protein and has been reported to play a role in fetal growth as well as growth of sarcomas.[36, 37] We report that *DNAJA4* promoter hypermethylation across 14 CpG sites was negatively associated with neonatal leptin levels, a measure of adiposity. The CpG region identified in our study overlaps with a region previously reported by Roifman, et al in a twin study of placental *DNAJA4* methylation and severe growth discordance.[34] While the direction of aberrant methylation and the tissue in which methylation was measured differed from our study, our replication of the same CpG region highlights that this gene region may play a role in the regulation of fetal growth. Our study is the first to report a relationship between cord blood *DNAJA4* and leptin, and thus further study into *DNAJA4* methylation patterns as they relate to fetal fat tissue accumulation and adipocyte leptin production are necessary.

We also found a positive association of *TFR2* hypermethylation and cord blood leptin levels in a region of the gene with multiple overlapping regulatory elements. Given the proximity to regulatory elements, methylation changes in this area may impact gene expression; however, expression analyses are necessary to fully evaluate this possibility. *TFR2*, expressed primarily in the liver, encodes transferrin receptor 2 which functions as a mediator of cellular uptake of transferrin bound iron. Iron homeostasis has been previously associated with adiposity, perhaps mediated by obesity induced inflammation.[38] In one study of mice fed high fat diets, *TFR2* was more highly expressed in adipose tissue compared to mice fed a usual diet.[35] However, the direction of our findings differ from this report, as in our study, increased *TFR2* methylation was positively associated with leptin levels. Furthermore, our study is the first to associate *TFR2* methylation with a marker of adiposity in a human population. *TFR2* may play a role in the regulation of adipose tissue but additional study is needed to fully elucidate these mechanisms.

Using ENCODE, we found multiple regulatory elements overlapping with the *DNAJA4* and *TFR2* DMRs, suggesting that the significant DMRs are in areas of active gene regulation (Figs 1 and 2). The potential for clinical impact due to altered methylation in these areas is physiologically plausible but without gene expression analyses, we cannot confirm or refute this possibility.

The genes identified in this study are novel in comparison to prior studies of newborn epigenetics and body size. Our previously unreported findings may be accounted for by the fact that the field of newborn epigenetics continues to emerge and evolve and methylation patterns are both tissue and age dependent. Furthermore, this study is not directly comparable to the majority of studies examining newborn methylation and childhood obesity as prior studies have largely focused on outcomes such as birth weight [14, 39] as opposed to anthropometric measures or biomarkers of adiposity or childhood body composition.[11, 12, 40] Pan et al’s study [13] had a similar aim as ours and identified *HIF3A* methylation in cord blood to be associated with newborn adiposity. While previously reported genes did not emerge in the current study, the genes we did identify were all implicated in tissue growth, leptin expression or adipocyte development. We propose that altered methylation in these genes lead to impaired adipocyte tissue differentiation and/or growth, thus altering adipocyte mediated leptin production. With more adipose tissue growth, we expect higher leptin production, perhaps inducing a state of leptin resistance, which, in turn is associated with impaired insulin sensitivity, insulin signaling and satiety signaling. [41, 42] Together, these factors can impact energy homeostasis and body composition. While the mechanisms underlying the development of leptin resistance and impact on obesity development are still being elucidated, literature suggests that high leptin levels in obese states are associated with adverse metabolic risk.

Strengths of our study include the use of a cohort of healthy mothers with documented normal glucose tolerance allowing us to remove the well-known confounder of maternal gestational diabetes on fetal adiposity development. In addition, the determination of infant body fat utilized a validated, non-user dependent method contributing to the accuracy and precision of our measurements. Use of the Infinium Illumina 450K array allowed us to examine approximately half a million CpG sites across 99% of RefSeq genes within the genome and identify novel associations.[43] Finally, our DMR analysis helped improve statistical power for detecting weak associations, as neighboring CpGs with similar effects reinforce each other. CpGs have been suggested to not only function individually, but also as a group to impact gene expression.[44] It is also believed that DMRs can control cell-type specific transcriptional repression of an associated gene. *DMRcate* is a data-driven approach to identify DMRs that can remove the bias incurred from irregularly spaced methylation sites to identify DMRs, which is particularly useful with the design of the Illumina platform and more powerful than other algorithms in detecting DMR where CpG coverage is sparse.

One limitation of our study is our inability to determine the impact that the identified methylation changes have on gene expression due to lack of available RNA. Expression analysis in relevant tissues, such as adipose tissue, is necessary to study the biological plausibility of our reported findings. Our study did not reveal any associations between cord blood gene methylation and direct anthropometric measures of adiposity such as neonatal % body fat, fat mass, and birth weight. Potential explanations for our inability to observe a statistically significant association with newborn fat include our small sample size and inclusion of infants both to women with documented normal glucose tolerance, which removed the well-known confounder of fetal hyperinsulinism and gestational diabetes on newborn macrosomia. [45] However, we did identify epigenetic associations with cord blood leptin levels. In this cohort [46] and others,[47] leptin has been highly correlated with measures of newborn fat and higher levels may impair normal satiety signaling, energy expenditures, and promote the development of insulin resistance.[48] Therefore, the reported associations are notable findings that may reflect subtle, early life changes in a newborn’s metabolic state that may have long term impact on metabolic health. Furthermore, there is little published literature to suggest whether the gene relationships identified in this study play a role in adipose tissue accretion, long term obesity risk or have a significant clinical impact. However, the findings in the *DNAJA4*, *TFR2*, *PLAG1*, and *FGF1* genes do suggest a possible underlying physiological mechanism for adipocyte growth mediated by these genes. Additionally, the cross-sectional design of our study only allows us to report associations and we are thus unable to comment on causality or the long-term impact of our findings. Lastly, generalizability is limited given our small sample size and our sole inclusion of mothers with documented normal glucose tolerance during pregnancy.

This study is among the first to examine associations of individual CpG sites and differentially methylated regions in cord blood DNA with cord blood leptin, a marker of neonatal adiposity. In particular, *DNAJA4*, *TFR2*, *SMAD3*, *PLAG1*, *FGF1* and *HNF4A* methylation represent novel and possibly physiologically relevant markers of neonatal adiposity. While studies in adults suggest that methylation changes are the consequence of adiposity, the direction in early life and the impact of the *in-utero* environment is not well characterized. Further study in larger cohorts is necessary to reproduce these our reported findings, elicit how gene methylation is controlled and determine whether these methylation changes impact gene expression. Following a longitudinal cohort over time is also necessary to determine how specific methylation patterns at birth translate to body composition and metabolic risk in childhood and adolescence.

## Supporting information

S1 Table Differentially methylated CpG sites associated with cord blood leptin levels. ^Percent change in leptin for every 0.01 increase in methylation beta value. *SNP containing probes were identified per the Illumina 450K annotation document. Cross-hybridization probes were identified per prior published literature by Chen et al 2013, Epigenetics 8(2):203–209.
(PDF)

S2 Table Evaluated CpG sites in the *DNAJA4* and *TFR2* genes. ^Percent change in leptin for every 0.01 increase in methylation beta value. *CpG sites that are included in the differentially methylated region (DMR). Hg19 Human Assembly was used to provide DMR location.
(PDF)
