# Supplementary material for: Association of cord blood methylation with neonatal leptin: An epigenome wide association study
Source: PLoS One. 2019 Dec 18;14(12):e0226555. doi: 10.1371/journal.pone.0226555 (PMC6919608; doi:10.1371/journal.pone.0226555)
Supplement: S1 Table — ^Percent change in leptin for every 0.01 increase in methylation beta value. *SNP containing probes were identified per the Illumina 450K annotation document. Cross-hybridization probes were identified per prior published literature by Chen et al 2013, Epigenetics 8(2):203–209. (PDF) [file pone.0226555.s001.pdf]

Table S1: Differentially Methylated CpG Sites Associated with Cord Blood Leptin Levels

Gene	Chromosome	CpG Site	% Change in Leptin^	FDR	Comment*
MIR122	18	cg19312305	-12.37	0.0053	SNP containing probe (rs3760546)
RABIF	1	cg22388200	45.19	0.0200	
MRPL15	8	cg07335376	13.14	0.0200	
SMAD3	15	cg03480935	-8.08	0.0200	
MIR485;MIR453	14	cg21071540	-13.19	0.0200	SNP containing probe (rs79064704)
ITGB2	21	cg13315706	7.46	0.0200	
C2orf84	2	cg26250093	24.48	0.0200	
C1GALT1	7	cg22244135	27.50	0.0200	
SERINC4;C15orf63	15	cg26552774	29.73	0.0200	
CCDC155	19	cg04830471	-16.50	0.0200	
PLAG1	8	cg21448513	-9.44	0.0200	
HSD17B13	4	cg08815403	-12.80	0.0200	
UBE2U	1	cg22450138	-16.21	0.0200	
R3HCC1	8	cg00329472	-24.49	0.0222	
HDAC11	3	cg21810733	-8.68	0.0307	
SLC26A10	12	cg26124597	13.63	0.0307	
PATE4	11	cg27618216	-9.42	0.0307	
KIAA1026	1	cg24943198	-12.94	0.0307	
EIF4G1	3	cg08822118	-8.38	0.0307	
HNF4A	20	cg16121136	-10.54	0.0307	
UOX;DNASE2B	1	cg03622431	-16.79	0.0307	
GNG2	14	cg09261791	-15.34	0.0308	
CACNB2	10	cg12289251	12.94	0.0308	
CTXN3	5	cg20744943	-14.21	0.0344	
PLCH1	3	cg20215257	-13.61	0.0344	
DLGAP4	20	cg10401741	-13.40	0.0349	
ESRRG	1	cg10090568	-10.01	0.0349	
C10orf84	10	cg02483596	-42.39	0.0349	
DZIP1L	3	cg14775286	-8.95	0.0352	
ARHGAP30	1	cg03089651	37.89	0.0354	
C20orf160	20	cg12084436	-13.69	0.0354	
TM4SF4	3	cg13688966	-11.61	0.0354	
LINGO4	1	cg13563504	8.51	0.0354	
S100A7	1	cg02892624	-8.64	0.0354	Cross-hybridization probe
CLCN6;MTHFR	1	cg05228408	-7.41	0.0354	SNP containing probe (rs45504095)
PRG1	19	cg19272150	-7.99	0.0354	
TPD52L1	6	cg17864650	-11.82	0.0354	
SPOCK2	10	cg12623536	14.49	0.0354	
TNFAIP8L2	1	cg23612220	19.36	0.0354	
ARF5	7	cg12288726	9.85	0.0354	
C10orf58	10	cg09203802	-10.64	0.0354	
TPH1	11	cg08400935	-17.10	0.0354	
MIR654;MIR376C;MIR376A2	14	cg02047319	-12.59	0.0354	
AKNA	9	cg02619205	-6.59	0.0354	
IFI30	19	cg01485548	8.18	0.0354	
OR2W5	1	cg09638704	-9.59	0.0356	
SH3BP4	2	cg01854776	-27.20	0.0363	
KIAA1026	1	cg07370352	-9.01	0.0376	
TRPV1	17	cg25222319	-25.72	0.0402	
PRR5L	11	cg23783076	-16.40	0.0402	
CACNA1I	22	cg18070442	-14.15	0.0404	
TAAR3	6	cg10655144	11.60	0.0412	
PPEF2	4	cg18327952	-11.38	0.0412	
CD5L	1	cg11139878	-17.03	0.0412	
PRKCZ	1	cg00866690	-24.88	0.0412	
GUCA1A	6	cg20927649	-9.81	0.0412	
PANK1	10	cg25770176	-7.35	0.0412	SNP containing probe (rs35260809)
ANXA2	15	cg22365276	20.46	0.0412	

PAPOLB;RADIL	7	cg07856421	-29.40	0.0412	
MORC1	3	cg05148217	-40.21	0.0412	
FGF1	5	cg13724550	-8.80	0.0412	
KIAA1688	8	cg06404526	-10.26	0.0412	
MIR299;MIR380	14	cg19603100	-16.91	0.0412	
CRTAC1	10	cg23801028	4.48	0.0415	
LINGO3	19	cg09370594	-37.88	0.0415	
ICAM2	17	cg12076102	-5.75	0.0415	
LOC643406	20	cg06182261	-11.36	0.0420	
MIR136;RTL1	14	cg04699566	-16.78	0.0433	
C5orf20	5	cg02379427	-9.99	0.0444	
PEMT	17	cg00520042	-17.90	0.0444	
GJB3	1	cg07999953	-17.18	0.0444	
CD53	1	cg16896205	-10.19	0.0446	
KIAA1671	22	cg02076598	-14.97	0.0446	Cross-hybridization probe
SH3BP4	2	cg01696784	-42.98	0.0446	
ANO1	11	cg24044957	-9.11	0.0446	
MINK1	17	cg18418479	-9.08	0.0446	Cross-hybridization probe
SLC6A19	5	cg17650028	-8.11	0.0446	SNP containing probe (rs67330201)
GPR156	3	cg08414647	-10.95	0.0446	
KIAA1671	22	cg26492686	-15.22	0.0446	Cross-hybridization probe
CSF2	5	cg02325250	-9.00	0.0446	SNP containing probe (rs77296537)
SRC	20	cg23410129	-18.81	0.0446	
RPRD2	1	cg08246527	34.36	0.0449	
TMIE	3	cg10775230	-11.52	0.0449	
MIR211;TRPM1	15	cg06314969	-11.31	0.0449	
RHBG	1	cg07702888	-7.48	0.0449	
PRKCZ	1	cg16653138	-41.84	0.0449	
OR10H1	19	cg16494597	-12.88	0.0449	Cross-hybridization probe
PRSS38	1	cg03651877	-32.23	0.0451	
SOHLH2	13	cg00208734	-26.15	0.0451	
RPL23A;SNORD4A	17	cg15036326	-11.99	0.0451	
HRH2	5	cg02389195	-11.01	0.0451	
ZNF238	1	cg16399365	22.91	0.0451	
CREB5	7	cg02124565	-11.67	0.0451	
WISP1	8	cg02903822	-20.45	0.0451	
NFYC	1	cg15986668	-5.43	0.0451	
CMIP	16	cg11419186	-8.48	0.0451	SNP containing probe (rs77588737)
PUF60	8	cg21639273	1516.31	0.0451	
RFFL	17	cg17036418	16.61	0.0451	
ADRA1D	20	cg18435870	17.50	0.0451	
GZMA	5	cg26357596	14.18	0.0451	
HOXC9	12	cg07634179	21.05	0.0451	
NUMA1	11	cg17997463	33.98	0.0451	
HDDC3;UNC45A	15	cg19670290	-9.12	0.0451	
LOC338799;SETD1B	12	cg21968169	22.35	0.0451	
NRAP	10	cg04712892	-15.16	0.0451	
IRF1	5	cg25410123	72.58	0.0451	
LOC388946	2	cg20705236	-12.73	0.0451	
AIG1	6	cg11314042	5.15	0.0451	
GFRA4	20	cg06919203	6.75	0.0451	
FXYD6	11	cg26047127	-14.36	0.0461	SNP containing probe (rs76936143)
ZNF160	19	cg08470053	20.00	0.0461	
BLCAP	20	cg24890964	-6.73	0.0461	
C10orf47	10	cg06440958	25.03	0.0461	
MYO1G	7	cg06787669	46.99	0.0461	
PRKCA;MIR634	17	cg20152382	-14.11	0.0461	
PROZ	13	cg04850479	-9.63	0.0461	
MS4A8B	11	cg10891482	-6.47	0.0461	
TAS2R40	7	cg24780236	-14.58	0.0461	
CBLC	19	cg21211730	-10.32	0.0461	

RHO	3	cg13180098	-9.03	0.0461	
SPATA22	17	cg06862644	-13.79	0.0461	
OSBPL9	1	cg10201328	28.92	0.0461	
TNXB	6	cg10993085	9.08	0.0461	
MIR642;GIPR	19	cg02452310	23.74	0.0461	SNP containing probe (rs13306398)
MIR451;MIR144	17	cg08204050	-5.65	0.0461	
PNPLA1	6	cg01976992	24.25	0.0461	
MEST	7	cg20380768	-10.63	0.0463	
A2BP1	16	cg00962147	-8.98	0.0463	SNP containing probe (rs74647143)
ARHGAP9	12	cg08339023	16.77	0.0463	
FBXL19;ORAI3	16	cg07599144	8.28	0.0463	
TMPRSS12	12	cg15169829	3.95	0.0463	
CARD14	17	cg20623503	9.93	0.0463	
AIFM2	10	cg14128415	33.71	0.0463	Cross-hybridization probe
PLCZ1;CAPZA3;	12	cg06233731	-14.15	0.0463	
TRPV2	17	cg16732648	-14.40	0.0463	
RAB27A	15	cg07707586	-14.60	0.0463	
TMEM135	11	cg17980364	-7.30	0.0463	
AMPD2	1	cg15447512	7.06	0.0463	
DKFZP686I15217	6	cg01454349	-7.76	0.0463	
DLGAP2	8	cg13725636	-13.20	0.0463	
ATP1A4	1	cg06956052	-8.07	0.0463	
ALKBH1;C14orf156	14	cg20385229	-6.49	0.0463	
TRIM75	4	cg03505125	-14.22	0.0466	Cross-hybridization probe
LOC400931	22	cg24731441	-5.82	0.0467	
CALHM3	10	cg25937598	-11.52	0.0467	
C10orf107	10	cg11914795	-12.74	0.0469	
SPATA8	15	cg02423618	-6.31	0.0471	
FBXO32	8	cg12897164	-5.59	0.0473	
TMEM40	3	cg24290948	-7.43	0.0478	
TMEM150C	4	cg23095451	-9.71	0.0478	
FBXL19;ORAI3	16	cg06084952	10.93	0.0478	
PITPNA	17	cg20426671	-8.11	0.0478	
DPEP2	16	cg10096215	8.23	0.0478	
GJA8	1	cg11385094	-7.70	0.0478	
TM9SF4	20	cg20672708	-10.33	0.0478	
PKHD1L1	8	cg15974931	5.96	0.0478	
AQP12A	2	cg22860848	-7.31	0.0478	Cross-hybridization probe
PCGF3	4	cg10843276	-6.12	0.0478	
SEPT9;	17	cg24576026	-23.56	0.0478	
DNAJB2	2	cg13388731	-5.65	0.0478	
LOC285205	3	cg05842169	-18.03	0.0478	
OR2G6	1	cg05943574	-14.77	0.0478	
TBC1D10C	11	cg20826709	23.21	0.0478	
CCL27	9	cg13777609	-7.95	0.0478	
PAG1	8	cg22562591	8.07	0.0478	
USP6	17	cg12954718	-8.30	0.0478	Cross-hybridization probe
LILRA1	19	cg15701210	-8.07	0.0478	
FGF2	4	cg03941587	9.56	0.0478	
MGAM	7	cg18971054	-12.65	0.0478	
AAAS	12	cg23032316	12.08	0.0478	
PTPRE	10	cg22568703	-14.71	0.0478	
FIGNL2	12	cg02420617	-8.26	0.0478	
SPTBN1	2	cg20872981	-9.67	0.0478	
ISLR	15	cg24779381	9.66	0.0478	
ELOVL2	6	cg25151806	10.46	0.0478	
C16orf45	16	cg16762408	-16.96	0.0478	
SHC4;EID1	15	cg09689279	34.22	0.0478	
PDZRN4	12	cg03842440	-11.10	0.0478	
MPST	22	cg17575915	19.32	0.0478	SNP containing probe (rs35156365)
ROBO4	11	cg03572680	-12.77	0.0478	
ASCC2	22	cg18253910	-8.89	0.0478	

KLK2	19	cg19582822	-8.53	0.0478	
PLCH1	3	cg01671681	-11.06	0.0478	
SLC6A19	5	cg02389859	-5.55	0.0478	
RPH3AL	17	cg25374161	-8.86	0.0478	
LOC100130017	4	cg05649126	-17.82	0.0478	
C1orf49	1	cg22430565	-7.75	0.0478	
RHAG	6	cg19862344	-9.22	0.0478	SNP containing probe (rs3757200)
MYH10	17	cg25930644	-7.69	0.0478	
MTMR11	1	cg13476072	-6.31	0.0478	
KPRP	1	cg11950860	-9.57	0.0478	
C10orf108;DIP2C	10	cg11223286	-7.23	0.0478	SNP containing probe (rs71482854), Cross-hybridization probe
C10orf99	10	cg07798731	-11.49	0.0478	
OR6W1P	7	cg11068352	-15.18	0.0478	
TOP1;PRO0628	20	cg06573902	31.76	0.0478	
ZNF697	1	cg27182150	-11.59	0.0478	
C2orf54	2	cg20140201	-11.30	0.0478	
EXPH5	11	cg23322223	-12.30	0.0478	
TMEM71	8	cg20955688	7.65	0.0478	SNP containing probe (rs11381545)
MIR1289-2;FSTL4	5	cg08360457	-8.62	0.0478	
AGPAT4	6	cg20352108	-15.16	0.0478	
NACAP1	8	cg23963995	-17.71	0.0478	
OR10J3	1	cg19378892	-20.49	0.0478	
NLRP3	1	cg07313373	40.63	0.0478	
C19orf43	19	cg05241586	14.78	0.0478	
KANK2	19	cg07888912	11.31	0.0478	
CSRP3	11	cg25619586	-14.03	0.0478	SNP containing probe (rs12222160)
HRCT1	9	cg02258201	-9.19	0.0478	
SLC6A6	3	cg14772599	-9.94	0.0478	
SNX18	5	cg26118943	5.95	0.0478	
MIR431;RTL1;MIR433	14	cg02022380	-9.62	0.0478	SNP containing probe (rs73349352)
TNNC2	20	cg17038577	-11.34	0.0478	
PIK3R5	17	cg07335619	-12.35	0.0478	
TTBK2	15	cg21579239	-5.68	0.0478	
NEDD4L	18	cg23757461	11.81	0.0478	SNP containing probe (rs186034)
CTCFL	20	cg25397426	-11.79	0.0478	SNP containing probe (rs73625090)
TRIM10	6	cg15109860	-6.14	0.0478	
ALDH3B1	11	cg01577760	-4.14	0.0478	
ACMSD	2	cg02812142	-10.52	0.0478	
LRRC30	18	cg03759556	-32.43	0.0478	
OR1K1	9	cg14108527	-11.73	0.0478	
PRKG1;MIR605	10	cg11478495	-13.55	0.0479	
MIR889;MIR539	14	cg18604419	-17.86	0.0480	
MYT1L	2	cg02179341	-17.39	0.0480	
TYSND1	10	cg04439776	10.41	0.0481	
EEF1D	8	cg26073101	10.28	0.0488	
FLJ12825	12	cg16165803	-25.32	0.0491	
ESR1	6	cg08907436	-18.17	0.0492	
ALDH3B1	11	cg09982224	-5.32	0.0492	
C15orf17	15	cg18183774	20.55	0.0492	
LYSMD1;SCNM1	1	cg07429591	23.54	0.0492	
LIMCH1	4	cg02951847	-13.71	0.0492	
VNN2	6	cg17836145	19.38	0.0492	
ARHGEF7	13	cg15011899	7.00	0.0492	
IL22RA2	6	cg23564241	-12.80	0.0492	
OR6P1	1	cg00160262	-13.14	0.0492	
FAM65C	20	cg16326902	7.50	0.0492	
MYO7B	2	cg07040894	-6.45	0.0492	
MIR1910;C16orf74	16	cg07829693	-7.47	0.0492	
CYB5R3	22	cg01194538	-5.81	0.0492	
SPINLW1	20	cg10379687	-10.61	0.0498	

SETBP1	18	cg16705273	-10.62	0.0498	
GRB7	17	cg14575854	3.65	0.0498	
FIGN	2	cg18834375	4.24	0.0498	
DDX4	5	cg09637885	-13.71	0.0498	
SLC4A1	17	cg02998129	-5.34	0.0498	
TNF	6	cg21370522	5.02	0.0498	SNP containing probe (rs41297589)

^Percent change in leptin for every 0.01 increase in methylation beta value

*SNP containing probes were identified per the Illumina 450K annotation document. Cross-hybridization probes were identified per prior published literature by Chen et al 2013, Epigenetics 8(2):203-209
