# Supplementary material for: Association of cord blood methylation with neonatal leptin: An epigenome wide association study
Source: PLoS One. 2019 Dec 18;14(12):e0226555. doi: 10.1371/journal.pone.0226555 (PMC6919608; doi:10.1371/journal.pone.0226555)
Supplement: S2 Table — ^Percent change in leptin for every 0.01 increase in methylation beta value. *CpG sites that are included in the differentially methylated region (DMR). Hg19 Human Assembly was used to provide DMR location. (PDF) [file pone.0226555.s002.pdf]

Table S2: Evaluated CpG sites in the *DNAJA4* and *TFR2* genes

CpG Site	Location	% Change in Leptin^ (95% CI)	FDR
DNAJA4			
cg07393586	78538539	-7.55 (-15.98, 1.72)	0.329
cg01324349	78538784	6.85 (0.43, 13.69)	0.207
cg13634653	78552612	14.31 (-5.21, 37.85)	0.388
cg04975272*	78555907	-4.89 (-7.47, -2.24)	0.034
cg04963153*	78555966	-4.47 (-6.70, -2.20)	0.027
cg07061145*	78556178	-9.94 (-16.14, -3.28)	0.080
cg02073525*	78556423	-3.95 (-6.18, -1.67)	0.043
cg23366752*	78556502	-3.33 (-5.38, -1.23)	0.061
cg08594606*	78556512	-4.70 (-7.27, -2.05)	0.039
cg09214398*	78556534	-4.35 (-7.13, -1.48)	0.073
cg22935921*	78556834	5.90 (-2.66, 15.22)	0.407
cg05392364*	78556860	8.93 (-7.68, 28.53)	0.499
cg16358679*	78556935	60.09 (9.24, 134.62)	0.145
cg17246382*	78556940	48.44 (-23.61, 188.45)	0.455
cg01786994*	78556945	60.19 (9.79, 133.71)	0.140
cg11019923*	78557532	-5.78 (-9.80, -1.58)	0.105
cg25025992*	78557584	-6.41 (-9.44, -3.27)	0.025
cg21642988	78558406	-3.86 (-7.55, -0.02)	0.236
cg27093605	78560514	3.53 (-3.18, 10.70)	0.499
cg12366279	78578444	16.51 (0.12, 35.58)	0.235
TFR2			
cg04002480	100218081	-6.82 (-13.56, 0.46)	0.268
cg05602715	100218455	-2.01 (-7.62, 3.93)	0.591
cg17398057	100218512	34.91 (0.58, 80.97)	0.229
cg05786630	100218568	17.66 (-14.15, 61.26)	0.500
cg01338494	100218617	18.33 (2.95, 36.01)	0.153
cg22337169	100218717	43.78 (-26.02, 179.43)	0.483
cg03808577	100219429	2.20 (-4.69, 9.58)	0.607
cg07956434	100220005	6.21 (-1.33, 14.32)	0.330
cg18224793	100222124	-1.50 (-6.32, 3.56)	0.611
cg08571362	100224015	-4.22 (-7.60, -0.71)	0.156
cg11432630	100224149	-1.46 (-5.87, 3.16)	0.602
cg19767562	100224437	-1.46 (-6.57, 3.92)	0.623
cg25985683	100224470	-4.72 (-23.65, 18.89)	0.649
cg11751434	100224557	3.21 (-2.48, 9.24)	0.477
cg15510373	100224934	4.62 (-7.89, 18.83)	0.586
cg09369818	100225006	132.62 (9.90, 392.33)	0.184
cg26104384	100227091	-2.61 (-8.62, 3.79)	0.554
cg06582411	100229865	3.31 (-0.54, 7.32)	0.310
cg04739306*	100230781	8.22 (3.31, 13.37)	0.045
cg01749347*	100230985	4.68 (1.81, 7.62)	0.051
cg04499151*	100231278	8.07 (2.10, 14.39)	0.105
cg15603896*	100231672	5.86 (3.54, 8.23)	0.012
cg06911630	100234123	3.81 (-7.45, 16.45)	0.599
cg12792367	100238750	0.76 (-2.39, 4.01)	0.641
cg10681065	100239172	-0.46 (-3.83, 3.03)	0.685
cg04423314	100239188	-1.36 (-4.75, 2.15)	0.568
cg02286663	100239307	-0.86 (-4.65, 3.07)	0.647
cg01919768	100239764	-0.41 (-3.96, 3.26)	0.691
cg16628205	100240094	-1.24 (-5.14, 2.81)	0.607
cg26369382	100240341	-2.47 (-6.01, 1.20)	0.409

^Percent change in leptin for every 0.01 increase in methylation beta value

*CpG sites that are included in the differentially methylated region (DMR)

hg19 Human Assembly was used to provide DMR location
